# Partnering with patients to get better outcomes with chimeric antigen receptor T-cell therapy: towards engagement of patients in early phase trials

**DOI:** 10.1186/s40900-020-00230-5

**Published:** 2020-10-14

**Authors:** Madison Foster, Dean A Fergusson, Terry Hawrysh, Justin Presseau, Natasha Kekre, Stuart Schwartz, Gisell Castillo, Sarah Asad, Grace Fox, Harold Atkins, Kednapa Thavorn, Joshua Montroy, Robert A Holt, Zarah Monfaredi, Manoj M Lalu

**Affiliations:** 1 Clinical Epidemiology Program, Blueprint Translational Research Group, Ottawa Hospital Research Institute, 501 Smyth Road, Ottawa, ON K1H 8L6 Canada; 2 School of Epidemiology and Public Health, University of Ottawa, 451 Smyth Road, Ottawa, ON K1H 8M5 Canada; 3 Patient Partner, Ottawa, ON Canada; 4 School of Psychology, University of Ottawa, 136 Jean-Jacques Lussier, Vanier Hall, Ottawa, ON K1N 6N5 Canada; 5 Blood and Marrow Transplant Program, The Ottawa Hospital, 501 Smyth Road, Ottawa, ON K1H 8L6 Canada; 6 Institute for Clinical and Evaluative Sciences, ICES uOttawa, 1053 Carling Ave, Ottawa, ON K1Y 4E9 Canada; 7 BC Cancer Genome Sciences Centre, Canada’s Michael Smith Genome Sciences Centre, 570 W 7th Ave, Vancouver, BC V5Z 4S6 Canada; 8 SPOR Program Facilitator, Ottawa Methods Centre, Ottawa Hospital Research Institute, 501 Smyth Road, Ottawa, ON K1H 8L6 Canada; 9 Department of Anesthesiology, The Ottawa Hospital, 501 Smyth Road, Ottawa, ON K1H 8L6 Canada; 10 Department of Anesthesiology and Pain Medicine, University of Ottawa, 501 Smyth Road, Ottawa, ON K1H 8L6 Canada

**Keywords:** Patient partners, Patient engagement, Patient and public involvement, PPI, Clinical trial

## Abstract

**Aim:**

Though patient engagement in clinical research is growing, recent reports suggest few clinical trials report on such activities. To address this gap, we describe our approach to patient engagement in the development of a clinical trial protocol to assess a new immunotherapy for blood cancer (chimeric antigen receptor T-cell therapy, CAR-T cell therapy).

**Methods:**

Our team developed a clinical trial protocol by working with patient partners from inception. Two patient partners with lived blood cancer experience were identified through referrals from our team’s professional network and patient organization contacts. Our patient partners were onboarded to the team and engaged in several studies conducted to develop the clinical trial protocol, including a systematic review of the existing literature on the therapy, patient interviews and a survey to obtain perspectives on barriers and enablers to participating in the trial, an early economic analysis, and a retrospective cohort study.

**Results:**

Engaging patient partners enhanced our research in ways that would not have otherwise occurred. By selecting patient important outcomes for data collection, our partners helped flag that quality of life and health utility measures have not been reported in previous CAR-T cell therapy trials for blood cancer. Our partners also co-developed a non-technical summary of the systematic review that summarized results in an accessible manner. Our patient partners reviewed interview and survey questions, to improve the language and appropriateness; provided recruitment suggestions; and provided a patient perspective on the results, thereby confirming the importance of findings. Input was also obtained on costs for the early economic analysis. Our patient partners identified costs that may be a burden to both patients and caregivers during a trial and helped to confirm that the overall structure of the economic model reflected the patient care pathway. Our patient partners also shared their diagnosis and treatment stories, which helped to provide the research team with insight into this experience.

**Conclusions:**

Contributions by our patient partners were invaluable to each component study, as well as the overall development of the trial protocol. We plan to use this approach in the future in order to meaningfully engage patients in the development of other clinical trials; we also hope that by reporting our methods this will help other research teams to do the same.

**Trial registration:**

Affiliated with the development of NCT03765177.

## Plain English summary

Patients (including informal caregivers like friends and family) can help to incorporate the patient perspective into research. Patient engagement can lead to identifying new study questions and outcomes, improving the appropriateness of a study’s methods and documents, and expanding the reach of the results. Despite these advantages, a recent report found that very few clinical trials report on patient engagement activities.

Our team recently involved patients in the development of a clinical trial. Two patients living with blood cancer partnered with scientists to plan a clinical trial for a new cancer therapy (“CAR-T cell therapy”). The patient partners were involved in several activities to support the clinical trial including: selection of outcomes for a systematic review of the literature, co-development of a non-technical summary of the review, suggestions for recruitment to a patient interview study, providing a patient perspective on the interview questions and results, providing input on the costs patients may face during a trial for an early economic analysis, and providing feedback on the trial informed consent document.

Contributions by our patient partners improved our research and the clinical trial protocol. They identified that outcomes important to patients are not currently reported in CAR-T therapy trials, highlighting the importance of working with patients early in study development. Our patient partners were also vital in improving the language of patient-facing documents, identifying patient and caregiver costs that should be considered in the early economic analysis, and helping to confirm interview study findings. In this article, we share our approach and suggest ways of moving towards more meaningful patient engagement in early phase clinical trials.

## Background

Patient engagement, the involvement of patients, caregivers, friends and family members in the research process, has numerous proposed benefits [1–5]. Partnering with patients at the beginning of a study to identify research gaps or priorities may help to improve the relevance of research findings, leading to greater research impact. As experts of their lived healthcare experiences, patients and their caregivers can also provide valuable insight into potential barriers and enablers faced throughout the care pathway and during research. Patients can provide crucial insight on ethical issues, such as providing reassurance that the study is appropriate, and feedback on how to handle difficult discussions, such as approaching and recruiting patients at sensitive times or for sensitive topics [6, 7]. Recent reviews indicate patient engagement is gaining traction in clinical research [8, 9]. Several national organizations, such as the Patient-Centered Outcomes Research Institute (PCORI, USA), the Strategy for Patient-Oriented Research (SPOR, Canada), and INVOLVE (UK) now provide support to teams through various means such as funding and infrastructure [1, 10, 11]. A recent systematic review has also shown that patient engagement is associated with modestly improved clinical trial recruitment [12]. Furthermore, it’s suggested that patient and caregiver partners may help to ensure research findings are communicated in an appropriate manner and improve dissemination of results in traditional and novel channels [5]. In addition to these benefits, it has been argued that an ethical obligation underlies the need to involve patients as they are the “ultimate end-users” of the knowledge generated, and in the case of publicly funded research, patients may want to ensure resources are allocated to areas felt to be of priority [5, 13].

Despite growing support and interest to involve patients as partners in research, and the significant potential for added value, previous reviews reveal gaps in the literature. For instance, a 2014 review identified that patient engagement activities often involve passive methods of engagement (e.g. focus groups, interviews, surveys versus deliberation/organizational participation), and often occur only at the beginning of studies [14]. Another recent systematic review by members of our team assessed engagement specifically in clinical trials and found that relatively few trials reported on patient engagement activities (only 23 trials) [9]. While it is important to remember that there is no singular approach to engaging patients in research, it is crucial for researchers to report on their approaches to patient engagement as well as the lessons learned, so that others may build upon these approaches and address any identified challenges.

### The getting better outcomes with chimeric antigen receptor T-cell therapy (GO-CART) program

Chimeric antigen receptor (CAR) T-cell therapy, is a promising new class of personalized treatment for blood cancers, where some of a patient’s own T-cells are removed, genetically modified in a laboratory to specifically target and kill cancer cells, then re-administered to the patient. Despite CAR-T therapy’s promising initial results for CD19 positive blood cancers [15], further work remains to confirm both the safety and efficacy of specific, novel CAR-T therapy products. Our team is thus conducting the first Canadian-led, early phase clinical trial to assess the safety and effectiveness of a novel CAR-T treatment for resistant blood cancer (Trial Name: Canadian-Led Immunotherapies in Cancer Trial-01 (CLIC-01); clinicaltrials.gov registration #NCT03765177).

Given the existing evidence that patient partners may improve the development and conduct of clinical trials, our team applied a novel integrated knowledge translation (iKT) approach to engage patients (from inception) in the development of a CAR-T cell therapy phase I/II clinical trial protocol. An iKT approach, where patients joined the team as equal partners (see Methods for further details), was chosen to ensure patients were meaningfully and actively engaged throughout the research program, while avoiding tokenistic engagement (“false appearance of inclusiveness”) [14].

### Aim

We aimed to partner with patients on each component and stage of clinical trial protocol development, and to better align clinical trial processes and resources with patient needs. This paper addresses the above noted gaps in the literature by describing our team’s approach, our lessons learned throughout this process, and ways that we hope to improve our program in the future.

## Methods

This manuscript has been reported according to the Guidance for Reporting Involvement of Patients and the Public (GRIPP2) reporting checklist (Additional file 1) [16]. For the purpose of the GO-CART program, we used the Canadian Institutes of Health Research’s (CIHR) definition of integrated knowledge translation, and as per Fergusson et al.’s review [9], we have used the Strategy for Patient-Oriented Research (SPOR) definitions of patient and patient engagement:

- *Integrated Knowledge Translation (iKT)*: “The central premise of iKT is that involving knowledge users as equal partners alongside researchers will lead to research that is more relevant to, and more likely to be useful to, the knowledge users. Each stage in the research process is an opportunity for significant collaboration with knowledge users, including the development or refinement of the research questions, selection of the methodology, data collection and tools development, selection of outcome measures, interpretation of the findings, crafting of the message and dissemination of the results.” [17](*Note: references from within the quote were removed for clarity.)
- *Patient:* “Individuals with personal experience of a health issue and informal caregivers, including family, and friends.” [18]
- *Patient Engagement:* “Meaningful and active collaboration in governance, priority setting, conducting research and knowledge translation.” [18]

### The Excelerator framework

**Fig. 1:**
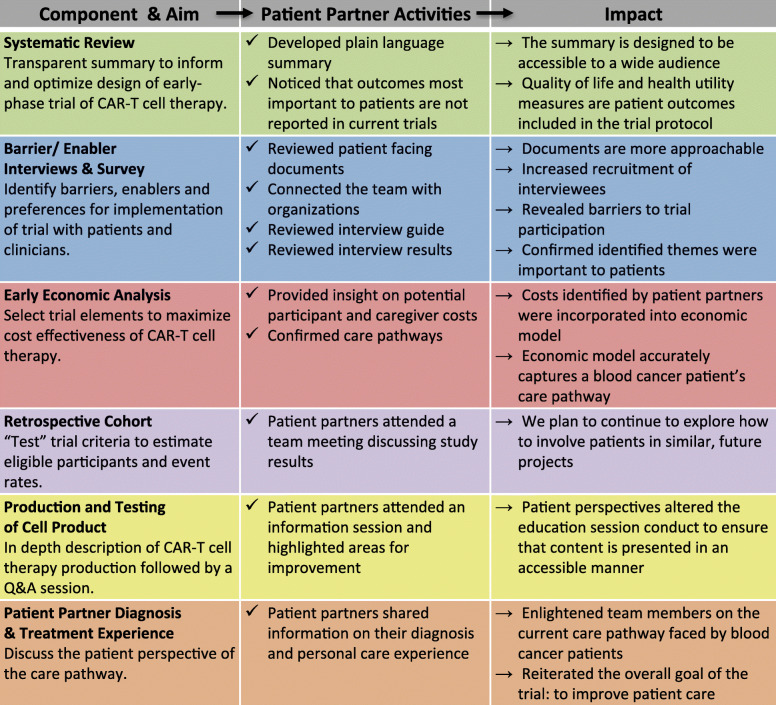
The Excelerator Framework. Our team developed a novel framework, involving five component studies (plus sharing of the Patient Partner Diagnosis & Treatment Experience), to evidence-inform an early phase clinical trial. Through application of our framework and use of an integrated knowledge translation approach we aim to accelerate the translation of CAR-T cell therapy to the clinic

As described in Fig. 1, our team developed a novel iKT approach involving five core component projects to evidence-inform a clinical trial protocol, where patients were involved from inception. Research methods and patient engagement have been described here; a more detailed description of the component studies can be found elsewhere [15, 19, 20] (Castillo G, Lalu MM, Asad S, Foster M, Kekre N, Fergusson D, et al: So you want to engineer my immune system to fight blood cancer? Identifying barriers and enablers to participating in an early phase immunotherapy trial for blood cancers, in preparation; Castillo G, Lalu MM, Asad S, Foster M, Kekre N, Fergusson D, et al: So you want to give engineered T-cells to patients? Identifying barriers and enablers to screening patients for a Chimeric Antigen Receptor (CAR) T-cell therapy trial, in preparation). In brief, the five components included a systematic review of the existing literature, interviews and a survey study to clarify patient and clinician perspectives on barriers and enablers to participation in the trial and trial delivery, an early economic analysis to assess factors that may influence the cost of CAR-T cell therapy (to identify how to maximize economic feasibility), a retrospective cohort study to refine eligibility criteria and assess trial feasibility, and production and testing of the cell product.

### Patient partner & stakeholder recruitment

Our interdisciplinary team aimed to identify patients with lived experience of blood cancer and who were willing to provide their input on our research. To ensure patient partner participation was feasible, we did not limit to the eligibility criteria for receiving CAR-T therapy (e.g. resistant blood cancer that recurs after standard therapy), as patients experiencing a relapse are typically quite ill and need to focus on their care. Recruiting patient partners that represent the ultimate end users of CAR-T cell therapy is a challenge unique to early phase clinical trials given the patient population that is eligible to participate and the novelty of the therapy. Three strategies were used to identify and recruit patient partners; first, hematologists on our team invited patients who they thought might be interested in the project. Prospective patient partners were provided with a verbal description of the project, as well as an information sheet. The information sheet provided an overview of the Excelerator project and summarized requirements, including participation in meetings (1 h/month), review the evidence from their perspective, provide input on how the trial would be run, and identify patient specific concerns throughout all projects. One patient partner was identified through this strategy and decided to join the research team. As a secondary strategy, our team partnered with a blood cancer patient organization (the Leukemia and Lymphoma Society of Canada, LLSC) to circulate an advertisement to their Ontario chapters, as well as through their website and newsletter. We had no responses to this advertisement. As a third strategy we also reached out to our contact within the LLSC to ask for direct referrals. One patient partner was identified through this strategy.

Stakeholders in other areas (i.e. hematologists and those with regulatory expertise) were also recruited to join the team to provide expertise and perspectives on potential barriers and enablers to conducting the trial.

### Patient engagement approach

To foster an inclusive environment, and build a team where patients worked as equal partners, we first held an orientation session and several educational sessions. Both patient partners met with the lead researchers (investigators, MML, JP, KT) and planned clinical trial lead (NK), who provided an overview of the planned project and described each of the individual component studies. We also held educational sessions explaining CAR-T cell therapy (e.g. manufacturing process, mechanism of action, how it is administered) and early economic analysis methods. These meetings provided an opportunity for patient partners to ask questions and become familiar with key terms and scientific jargon that could come up throughout the course of the project. Patient partners met one-on-one with the investigators for the educational sessions to help ensure they felt comfortable to ask questions. Briefing sessions were conducted prior to key activities to provide context and necessary background information. Patient partners were able to meet with the project leads throughout the course of the study to discuss specific areas of interest, and a patient engagement lead (SA) to discuss study updates and progress. In-person meetings with the group were informal and group discussion and questions were heavily encouraged throughout the sessions to create a safe space to share ideas and beliefs. The research team kept in regular contact with patient partners throughout the project by email. In addition, we summarized progress through a newsletter, which was sent twice (mid-project and at the end of the project).

### Developing a terms of reference

With our patient partners (TH, SS), we co-developed a document outlining the ways our team planned to work together, also known as a ‘terms of reference’. Using the INVOLVE organization’s template [21] (a resource which can be used by teams to clearly outline roles and responsibilities of group members as well as the overall goals of the group or project), we outlined the planned curriculum for patient involvement throughout each of the five component projects, to help create a shared set of expectations for all team members (Supplemental files 2–3). Throughout the project, the team aimed to create a respectful environment where all members felt valued. This was demonstrated through provision of encouragement, support, and mentorship, and by inviting our patient partners to attend the monthly team meetings (remotely or in-person) and encouraging them to share their opinions and comments during presentations and discussions. Patient partners were also invited to provide input on each of the component projects based on interest and availability. Acknowledgement on publications, presentations, and other forms of dissemination were agreed upon. In addition, reimbursement for any costs of travel were discussed.

### Face-to-face meetings & conferences

Our patient partner (TH) had the opportunity to attend two annual meetings held at conferences hosted by our funder BioCanRx (2017 and 2018 BioCanRx Summit for Cancer Immunotherapy; BioCanRx is a Government of Canada funded Networks of Centres of Excellence). These annual face-to-face meetings helped to further foster our team’s collaborative nature. During the 2018 meeting, TH also participated as a patient scholar at the Cancer Stakeholder Alliance’s (CSA) Learning Institute, a program which provides interested patients with the opportunity to attend educational sessions and conference presentations. TH found this opportunity allowed for mutually beneficial discussions between patients, researchers and trainees.

## Results

Below we have described patient participation in each component study of the Excelerator approach.

### Systematic review

Patient partners (TH, SS) met with the research team members to provide input on outcome measures to be extracted from included studies. Our patient partners identified quality of life and health utility measures as important outcomes to patients. We found that of the 60 clinical trials identified by our systematic review, none reported these outcome measures [15]. Though we were unable to extract data for these outcomes, our finding points to the need for CAR-T cell therapy clinical trials to consult with patients during protocol development so that such outcomes can be measured and reported.

Patient partners (TH, SS) also worked alongside the research team to develop a non-technical (‘plain language’) summary of the final results; this summary has proven to be a valuable resource as it describes the current evidence available on CAR-T cell therapy in language accessible to a wide audience. We have also found this document to be useful in describing the rationale for our trial in other component studies of our Excelerator approach (e.g. patient survey described below).

### Barriers/enablers to trial delivery interviews

Patient partners (TH, SS) provided a letter of support to the Research Ethics Board (REB), reviewed patient-facing materials such as the recruitment information sheet, as well as the interview guide to provide feedback. Our patient partners helped to modify the language of these documents to ensure they were approachable and accessible. Patient partners also suggested additional questions that could help to identify potential barriers and enablers to patient participation in the trial and prompts to help with the flow of the interview.

When we encountered difficulty accruing a minimum number of participants for this interview study, our patient partner (TH) helped identify additional organizations to approach for recruitment. TH liaised with the LLSC and identified additional avenues to distribute our recruitment information, such as the Chronic Lymphocytic Leukemia Patient Advocacy Group (CLLPAG). Our connection with the CLLPAG led to a last-minute offer to advertise our study at a conference where over 200 patients would be in attendance; we were unable to capitalize on this opportunity however, as we were not able to obtain REB approval in time.

After completion of the interviews (including coding and anonymization of the data) our patient partner TH was involved in providing his outlook on key themes identified from the patient and hematologist interviews, confirming which were most relevant from a patient perspective. This was done through both a meeting with the research team where preliminary results were presented and discussed, as well as through TH’s review of a document outlining key themes and illustrative quotes. This process helped to shape the manuscript around the themes felt to be of most importance to patients.

### Barriers/enablers to trial delivery survey

One of our patient partners (TH) participated in a think-aloud session to pilot the survey, by providing comments on their interpretation of the questions, feasibility of the survey, and any technical issues. This allowed us to assess whether questions were interpreted appropriately, ensure that the survey was feasible, and make technical and aesthetic modifications prior to distribution.

### Early economic analysis

Our patient partner (TH) worked with the team’s health economists to provide input on various expenses that may be incurred by patients throughout their illness and which may impact trial participation. This was done so that the economic model captures patient care trajectory and related costs. Importantly, TH also pointed out costs that may be incurred by patients’ supporting family members and caregivers. Identified costs included travel, parking fees, lodging expenses, and loss of income. This collaboration allowed us to identify expenses that we would not have otherwise recognized. TH also provided his perspective on the expectation of CAR-T therapy efficacy, and as well confirmed that the overall structure of the economic model appropriately captured the care pathway of a patient with a hematologic malignancy [20].

### Retrospective cohort study

Though we were not able to identify a patient partner role throughout conduct of the retrospective cohort study, our patient partner attended a team meeting where the results from this project were discussed. In the future, we look forward to exploring how patient partners may be more actively involved in this type of study. We are currently developing other ways for patient partners to be included at this stage of the Excelerator program.

### Production & testing of cell product

Our patient partners participated in an information session where the head of production provided an in-depth description of production methods to share knowledge on how CAR-T cell therapy is developed and tested, as well as to answer questions. Due to the highly technical nature of the CAR-T cell product development, it was a challenge to ensure all members of our multidisicplinary team understood the complex processes involved. To address this challenge, we created a collaborative environment to encourage discussion and shared perspectives or concerns. As engagement throughout this component primarily focused on informing and information sharing, we hope to have continued discussions with our patient partners as to how we may more actively engage patients as partners in future, similar studies.

### Patient partner diagnosis and treatment experience

One of our patient partners (TH) shared their diagnosis and treatment story with the team. All members greatly appreciated TH sharing his diagnosis and care experience as this helped to enlighten the team on the typical care pathway faced by patients with blood cancers. Although the ultimate goal to improve patient care is always our main motivation, as researchers, we can sometimes get caught up in details, documentation and deadlines. Through sharing his experience, TH further strengthened the team’s motivation and brought our ultimate goal to the forefront. Hearing the patient perspective also highlighted the importance of ‘humanizing’ trial development at the earliest stages to ensure all downstream development of a therapy is patient centered.

### Expansion to a patient panel & preparation for the trial

As we prepared for the initiation of the clinical trial, we expanded our program to include a patient peer support panel. Recruitment for our interview study and networking at the BioCanRx Summit allowed for patients in the provincial and national communities respectively, to learn more about our program. This resulted in our team being approached by two patients who expressed interest in joining the team.

Individual components of the Excelerator studies were used to inform the clinical trial protocol and ensure that its development was evidence informed. In addition, our patient partners contributed to trial development by reviewing the protocol and providing input on multiple aspects (e.g. frequency of follow-up appointments, what should be communicated to enrolled participants). Our patient partners also provided input into the patient informed consent approach created by NK and the clinical team, including the informed consent form, visual consent aids and a non-technical summary. In order to aid these activities, our team submitted a successful grant application with our patient partners, which has enabled us to continue to expand, as well as host a face-to-face meeting with our patient partners.

### Dissemination of results

Both of our patient partners are co-authors of this manuscript. In addition, TH and MML shared experiences with the GO-CART program at several invited speaking engagements for patient organizations and funders seeking to establish similar programs.

## Discussion

Our GO-CART patient engagement program helped to improve the development of our trial protocol substantially. In addition to identifying important outcomes for measure in our upcoming trial, our patient partners helped to describe our systematic review findings in an accessible manner. Our patient partners provided guidance on how to improve our study materials, including the interview study recruitment document, interview guide, and survey, in addition to providing their perspective on the key findings. One of our patient partners also provided insight on important patient and caregiver expenses and helped to ensure that the early economic analysis model appropriately captures a blood cancer patient’s care pathway. Furthermore, development of a terms of reference helped to document how our team worked together. To our knowledge, this is one of relatively few blood cancer research programs wherein patient partners worked alongside the research team from inception to develop a trial protocol for an early phase clinical trial.

### Benefits of patient engagement in early phase clinical trials

Implementation of the GO-CART patient engagement program resulted in numerous benefits. Partnering with patients from inception and obtaining the patient perspective throughout our research program helped to improve each individual component study and the final clinical trial protocol. Most importantly, we believe working with our patient partners from inception of trial development will help improve the relevance of the clinical trial findings to patients. For instance, based on the outcomes identified by our patient partners we plan to include a quality of life measurement within our trial. The impact of a treatment on the quality of life of a patient is a key piece of information for both patients and oncologists, and yet its use remains relatively low in cancer trials [22]. Despite the early-phase nature of our trial, collecting quality of life information from participants may provide us with critical data moving forward.

We believe a key component to the success of our engagement program was engaging with patients early (at the inception of the project) to ensure our relationships were built on a strong foundation. This is consistent with research teams engaging stakeholders in other health areas such as depression and asthma. McConnell et al. reported that early engagement was beneficial in that it “promoted equal ownership” [23], while Supple et al. noted that engagement “is often most impactful in the project formation phase” [24]. Certainly, our own experience would support these previous observations, as we believe our early engagement of patient partners improved feasibility of incorporating their feedback, strengthened relationships, and thereby maximized impact of patient engagement.

We also found engaging our patient partners all the way through the research continuum (e.g. discussion of study results and co-production of manuscripts) to be a rewarding experience. While a review of clinical trials found that most trials involving patients do try to continue their initiatives across the research continuum, overall very few report engaging patients at all [9]. By working with our patient partner to identify key themes from the interview study we were able to ensure that the results we focused on were also felt to be of importance from a patient’s perspective. Additionally, by working with our patient partners to develop a non-technical summary of the systematic review findings we hope to have improved the accessibility of our findings to a wider audience. A recent study similarly reported that working with stakeholder partners throughout the analysis and dissemination phases of a project not only improved the format of the article but as well allowed for incorporation of “real-world interpretations” [23].

Our patient partners also found participating in this research project to be a rewarding experience. TH noted that being involved allowed him to learn more about “how things work” in clinical research, which he felt enabled him to provide more meaningful feedback. He also felt that being involved throughout the various stages of the project provided him with a sense of contribution to improving patient outcomes.

### Challenges & areas for improvement

A key challenge of this project was the identification of our patient partners, and in turn, difficulty in obtaining multiple and diverse perspectives. Though various recruitment methods were used, we were only able to recruit two patient partners. As we primarily recruited through a third party, it is unclear why we faced this challenge, however we speculate that a few factors may have contributed, such as patients needing to focus on their health care, lack of compensation for time, and timing of meetings. We aim to address the issue of compensation for time in future initiatives as described in detail below. We also speculate that patients may have been intimidated by the technical nature of some of the activities involved in developing an early phase clinical trial protocol (review of the existing evidence, early economic analysis). Though we aimed to address this issue by highlighting that no prior experience with clinical trials was needed in our advertisement, we recognize that further effort, such as working with patient partners to develop recruitment materials (e.g. testimonial video), will be required in future initiatives.

We recognize that a limitation of involving only two patients is the potential for bias towards personal views and experiences of a limited number of patient voices. As we start to expand to a larger patient panel, we aim to work towards involving more partners with diverse backgrounds and viewpoints. We also note that we did not engage with patient partners who themselves were eligible for CAR-T cell therapy. While this was done to ensure that engagement was feasible, we recognize that engagement of this population may have yielded different perspectives. To address this limitation, we attempted to gain input from patients who would be eligible for CAR-T cell therapy through our barriers/enablers to trial delivery interviews and survey, which allowed for short-term participation (one-hour phone or in-person interview, or an online survey). Furthermore, we were unable to recruit any caregiver partners, a group who our patient partners pointed out would additionally provide a meaningful and unique perspective. Challenges in recruiting have been reported by others in the literature [9, 14, 25]. A previous review aimed to characterize best methods to identify and recruit patient partners, however, limited reporting and lack of comparison studies resulted in few proposed solutions [14]. Our experience would suggest that targeted recruitment through direct referrals may be more successful than open calls for patient partners. Clearly, further work remains to determine best methods of practice for identification and recruitment of patient partners.

Another key challenge stemmed from the technical nature of early-phase clinical trials. Because the primary outcome of most early phase clinical trials is safety and feasibility, much of the focus is shifted towards the therapy and away from the clinical aspects. In our experience, it is clear that cell therapy development processes are complicated concepts to understand for individuals without a basic science background. Not only does this challenge patient partner engagement, but also that of all team members without basic science training. Although this is a clear limitation throughout this program, it has highlighted the need to explore avenues for patient engagement in basic science research.

When providing feedback on the program, our patient partner (TH) stressed the importance of training and provision of background information. Though TH felt the education and briefing sessions were well done, he suggested several potential areas for improvement including ensuring topics are covered at a manageable pace (e.g. parsed into several sessions), sending presentations, materials and a list of acronyms ahead of time to allow for partners to do their own reading and research, as well as some additional topics of interest. Another potential option may be to co-develop and produce educational sessions with a patient partner, as done by Bell et al. for a patient-oriented research curriculum [26]. TH also noted that reflection and iterative revision of the terms of reference, and consistently communicating how patient partner contributions were incorporated into the project (and any associated outcomes) are essential to providing a sense of accomplishment. These activities also help to highlight how contributions are valued. Although many of these goals were met, an increase in personnel dedicated to patient engagement in our program would have helped ensure these issues were consistently addressed.

We were unable to provide monetary compensation for our patient partners’ time; however, we were able to reimburse for all travel and accommodation fees incurred for meetings. These terms were discussed with our patient partners prior to beginning the project, as well as outlined in our terms of reference. For future projects, we are incorporating patient partner compensation into our grant application budgets, in accordance with recent guidance published by the Strategy for Patient-Oriented Research (SPOR) Networks in Chronic Diseases and Primary and Integrated Health Care Innovations (PICHI) Network [27] and the SPOR Evidence Alliance [28]. Upon asking for feedback from our patient partner, it was also noted that re-payment of expenses associated with travel tended to be slow from our academic institution. We aim to better address this concern by communicating reimbursement timelines to our patient partners, setting firm targets and connecting our patient partners with a research assistant who will help to expedite the process.

At various times throughout the program, our team (both researchers and patient partners) faced technical difficulties when using the teleconference software. This led to some frustration, however with time and practice our team was able to overcome this issue. Another challenge was that team meetings typically took place during usual business hours. As our patient partners both work full-time, our team greatly appreciated that they were willing to take the time to attend and understood that other priorities would sometimes prevent them from attending. We aimed to address this challenge by documenting discussions through meeting minutes and encouraging our patient partners to schedule a phone discussion with the lead investigator if they had any remaining questions or were interested in a recap of the discussion. Finally, while we had aimed to provide consistent updates through a newsletter, given the small number of patient partners engaged we ended up providing regular updates through email and felt that a more frequent newsletter would have been redundant. However, as the program expands to a larger patient panel we intend to send more consistent updates though a newsletter, which will also help to ensure patient partner contributions are summarized and shared with the team and a broader network of stakeholders.

## Conclusions

We believe this overview of our program helps to advance the existing literature on patient engagement. A previous review by members of our group found reporting of patient engagement details to be very limited in the clinical trial literature [9]. By documenting our program we hope to share our experience with other research teams so that they may learn from and build upon our work.

## Supplementary information

**Additional file 1.** GRIPP2 Reporting Checklist.

**Additional file 2.** Detailed Methods of Terms of Reference Development.

**Additional file 3.** Terms of Reference.

## Data Availability

Not applicable.

## Abbreviations

CAR-T: Chimeric antigen receptor T-cell therapy
CRS: Cytokine release syndrome
