# Supplementary material for: Partnering with patients to get better outcomes with chimeric antigen receptor T-cell therapy: towards engagement of patients in early phase trials
Source: Res Involv Engagem. 2020 Oct 14;6:61. doi: 10.1186/s40900-020-00230-5 (PMC7557015; doi:10.1186/s40900-020-00230-5)
Supplement: Supplementary file 2 — Additional file 2. Detailed Methods of Terms of Reference Development. [file 40900_2020_230_MOESM2_ESM.pdf]

Additional File 2: Detailed Methods of Terms of Reference Development

As per a recommendation from our local Ontario SPOR SUPPORT Unit (within the Ottawa Methods Centre, ZM), patient partners (TH, SS), the study co-investigators (MML, JP) and research assistants (SA, MF) developed a patient partner terms of reference document to describe how the team planned to work together. The primary goal was to create a working document to provide guidance and a shared set of expectations, however all elements were modifiable in that they could be updated to suit the teams' needs as time went on. Our terms of reference document was crafted using the template suggested by INVOLVE [20]. Key sections outlined within the terms of reference included the aims and responsibilities of the patient partner group, patient partner recruitment strategy, a key point of contact for patient partners, how the group planned to review the patient engagement program and terms of reference, the format of meetings, sharing of materials, reimbursement, and definitions of key terms.

The template was modified as appropriate; for instance, additional components suggested by our patient partners were added. TH recommended that a hierarchical map of reporting relationships be developed to clearly outline who was leading or involved in each project. Furthermore, sections felt to be irrelevant or unnecessary were not used. For example, our SPOR SUPPORT unit noted that other research teams have included signed, non-disclosure agreements, but encouraged that we proceed in a manner that was felt to be appropriate for our team. As we felt this component was not necessary, the team instead briefly discussed that materials and discussions should be treated as confidential and only shared within the group.
