# Supplementary material for: Partnering with patients to get better outcomes with chimeric antigen receptor T-cell therapy: towards engagement of patients in early phase trials
Source: Res Involv Engagem. 2020 Oct 14;6:61. doi: 10.1186/s40900-020-00230-5 (PMC7557015; doi:10.1186/s40900-020-00230-5)

**Additional File 3: Terms of Reference**

## GO-CART Patient Partner Terms of Reference

**Name of group:** Getting better Outcomes with Chimeric Antigen Receptor T-cell therapy (GO–CART) – Patient Partners Group

**Title:** Terms of Reference for Patient Partners (last updated March 2, 2018)

**Purpose/role of the group:**

- Objective of the GO-CART project:
  The GO-CART Excelerator program will address issues to inform a CAR-T cell trial protocol to maximize its overall feasibility, safety, effectiveness, and economic feasibility. The GO-CART program consists of five separate but related projects: 1) Preclinical & Clinical Systematic Reviews, 2) Knowledge Translation, 3) Early Economic Analysis, 4) Retrospective Cohort Study, 5) Production and testing of cell product. The ultimate aim of the project is to develop a protocol for a first-in-Canada clinical trial to evaluate safety and feasibility of administration of CAR-T therapy for hematologic (blood) cancers.

- Objective of the Patient Partners:
  To provide insight into each of the 5 projects (as per interest and availability) to inform the development of the trial protocol based on lived experience with hematologic cancers.

- When was the Patient Partners group established and by whom?
  The Patient Partners group was established in 2017 by Dr. Manoj Lalu and Dr. Dean Fergusson.

- What are the aims/responsibilities of the Patient Partner group?
  Provide support and insight based on lived experience to the following groups:
  - **Preclinical & Clinical Systematic Reviews**
    To date, Patient Partners have helped to develop a lay summary for the systematic review. Patient Partners will also help to clarify which outcomes are important to patients, to ensure the selected outcomes align with patient interests. When developing the systematic review protocol, Patient Partners identified efficacy, safety and quality of life measures as important outcomes.

  - **Knowledge Translation**
    Patient Partners can provide input on the study design (e.g. method of consent, recruitment strategies), recruitment materials (e.g. information sheet), data collection tool (e.g. interview guide), and data analysis (e.g. review some raw

interview data and discuss themes rising from participants' comments). Patient Partners will also be invited to review the survey draft and resulting analysis.

- **Early Economic Analysis**

  Patient Partners can help suggest important and meaningful outcomes to be captured in the economic analysis. Patient Partners may also help to provide feedback on the methods and study results. With close collaboration with the Patient Partners, we hope to disseminate clear and easy to understand key messages.

- **Retrospective Cohort Study**

  It will be helpful to receive input from Patient Partners on the typical treatment pathway for hematological cancers, so that the care pathway can be discussed and compared to potential changes upon implementation of CAR-T cell therapy.

- **Future trial related aims:**

  During preparation for the trial, Patient Partners can be involved in:

  - Writing letters of support to the Research Ethics Board and funding agencies
  - Reviewing consent forms for content and clarity
  - Contributing to the development of educational materials (i.e. multimedia aspects)
  - Reviewing the risks/benefits outlined in the consent forms and educational materials for clarity
  - Selection of outcomes and intensity of data collection (amount and timing of data collection)
  - Patient outreach to support those in the trial (i.e. answering questions)
  - ***Note:** This section will be reviewed and updated closer to the trial.

For future projects we will aim to involve Patient Partners in the early stages of group formation and initiative development.

**Patient Partnership Membership:**

**Recruitment Strategy**

- To date we have recruited Patient Partners via the Leukemia and Lymphoma Society of Canada (LLSC) as well as through The Ottawa Hospital.

- *What other ways will we use to recruit?*
  We can approach Cancer Care Ontario (CCO), Canadian Cancer Society Research Institute (CCSRI), The Leukemia & Lymphoma Society of Canada (LLSC), and Lymphoma Canada for future recruitment of Patient Partners. We can also recruit through team referrals.

- *Description of any changes to recruitment process:* None to date (March 2018).

- *Who is the membership of the group open to?*
    1) People with lived experience of hematologic cancers. We have not narrowed down to a particular type of cancer. For the future, we may also want to consider geographical representation across Canada for Patient Partners.
    2) Caregivers of individuals with lived experience of hematologic cancer.

- *Planned number of Patient Partners?*

    There are no restrictions on number of Patient Partners, but we will aim for 3-5 partners. We will expand to more people for the trial.

- *Are any representatives from other organizations included?*

    One of our Patient Partners was recruited through The Leukemia & Lymphoma Society of Canada (LLSC).

- *Orientation Session*

  Newly recruited Patient Partners will meet with at least one research team member for an orientation session. This will involve a discussion of the overall goal and components of the GO-CART project. Patient Partners may also request further one-on-one meetings with research staff members and project leaders if they would like more information on a specific topic.

- *How long is the period of Patient Partnership membership and can it be extended?*
    - *How many hours per month?*

      It is recommended to attend the 1-hour GO-CART meeting once a month.
      Ad-hoc involvement and meetings for the other specific projects will be based on the stage of the project and Patient Partner availability.
    - *How many months of involvement?*

      The project will continue until January 2019 and beyond that it would be great to have Patient Partners on board for the actual clinical trial (during 2019). Please feel free to decrease or stop participation at any point of the project.

**Accountability:**

- Madison Foster (Research Assistant) will be the main contact for the Patient Partners, however Manoj will be copied onto every communication for consistency. **Note:** Due to the nature of research teams, it is possible that research team members may join throughout the course of the project. This new person will be introduced at a team meeting, and their name and role will be added to the Appendix.

**Review:**

*How often will the group review the relevance and value of its work and the terms of reference?*

- We will review the terms of reference every 6 months (Dec and June) and in between if new team members join.
- For regular evaluation of our partnership: we will use an evaluation tool for guidance
- Patients Canada: http://ossu.ca/wp-content/uploads/EvaluationSurveysPatient_2016.pdf, http://ossu.ca/wp-content/uploads/EvaluationSurveysResearcher_2016.pdf
  - Please note that there are separate surveys for Researchers and Patients.
- Madison (Research Assistant) will provide feedback via a newsletter report or brief project summary 2-4 times per year. The report will include project updates and information on what was done and how Patient Partner feedback was taken into account (e.g. why some feedback was taken into account and why others were not).
- The terms of reference document will also be reviewed and updated prior to the clinical trial to reflect any new opportunities for participation and involvement for this phase of the project.

**Working methods / ways of working:**

- *Will any subgroups be convened?*
  - Each sub-project of GO-CART has a working group; there will be options for Patient Partner involvement in each subgroup.

- *Ways of working together*:

  **Meetings**:
  - *How many meetings will be held each year and where will they be held?*
    To date, this has varied based on when we needed input/insight from Patient Partners. Patient Partners are always included in the larger team meetings. Meetings will usually be held at The Ottawa Hospital, however members may also join by teleconference (for further details please see '*What will the format of meetings be?*').

  - *How will topics for the agenda be generated?*
    Topics are discussed during team meetings.

  - *How and when will meeting papers be circulated?*
    Meeting agendas are circulated in advance by Josh Montroy (Research Associate). For subgroups and minor group meetings, a research assistant or associate will communicate the agenda and relevant meeting information by email.

- *What will the format of meetings be?*
  Meetings are a combination of presentations and discussion in a group setting, typically around a table and lasting for one hour. It is possible to join meetings by teleconference, as well as to join via ‘Go To Meeting’, an online platform that allows participants to see what is presented onscreen virtually on their own computer. Use of ‘Go To Meeting’ requires a bit of practice (and sometimes technology can fail); the research team is on hand to provide support and guidance. At the beginning of each meeting an overview using lay terms will be given, to ensure understanding among all members. Our team strives to create a collegial and supportive environment, respectful of the fact that members will be coming from various backgrounds.

- *Will non-members be invited to group meetings and if so, under what circumstances?*
  Only group members have attended group meetings (this includes Patient Partners).

- *Who will provide secretariat for the group?*
  This rotates every meeting; it is a member of the research team.

**Sharing of information and resources (including confidential materials)**

- *How will group members share information and resources?*
  Information and resources have been primarily shared via e-mail. For data analysis, a Dropbox folder will be used to share large data sets.

- *Will there be a web space for the group and if so, will it be password protected and who will be responsible for facilitating it?*
  Currently, all online communication will take place through e-mail. Downstream, for the clinical trial, we intend to set-up a web space for the group. Closer to the trial we will designate a member of the research team to be responsible for facilitating it, and discuss whether the forum should be password protected. The terms of reference will be updated to reflect these changes.

- *How will confidential materials and copyright issues be identified and dealt with?*
  As meetings and study materials may involve intellectual property, we ask that all members of the team keep study materials, and discussions regarding confidential information, private (within the group).

  Should any specific confidentiality or copyright issues arise throughout the project, these will be discussed among the group at a team meeting. The terms of reference will be updated to reflect these issues. **Note:** This section should be re-visited prior to the clinical trial.

**Reimbursement of Expenses**

- Patient Partners will be reimbursed for all travel expenses incurred to attend meetings and group events. This includes parking and transportation (airfare, train, etc.).

**Definition of Terms**

An educational session was completed to go over the technical elements of the CAR-T cell therapy and its production. Patient Partners have also previously reviewed elements of the various projects in a one-on-one meeting with research staff members and project leaders. An orientation meeting will take place for any new Patient Partners, to discuss the overall goal and components of the GO-CART project. Patient Partners may also request further one-on-one meetings with research staff members and project leaders if they would like more information on a topic. At the beginning of each meeting, an overview using lay terms will be given to ensure understanding among all members. Below several key terms have been defined.

**CAR T-Cell Therapy:** CAR T-Cell Therapy involves genetically modifying a patient's own T-cells (a type of immune cell that can be collected from the blood) to target and kill cancer cells.

**Knowledge Translation:** Knowledge Translation is a field of research that involves strategies to ensure uptake of research findings into practice.

**Systematic Review:** A systematic review is a literature search, which aims to identify studies on a specific research question in a non-biased manner. This is done by following a structured protocol.

## APPENDIX: Team Members and Roles

| Team Member | Role |
|---|---|
| Terry Hawrysh | Integrated Knowledge User: Patient Partner |
| Stuart Schwartz | Integrated Knowledge User: Patient Partner |
| Manoj Lalu | Management of overall GO-CART project. Will specifically lead meetings, integrated knowledge translation efforts, and systematic reviews. |
| Dean Fergusson | Oversight of GO-CART project. Will specifically co-lead systematic reviews, prospective cohort study, and trial protocol development. |
| Natasha Kekre | Clinical lead for the GO-CART project. Will advise on all components and specifically lead the retrospective and prospective cohort studies. |
| Justin Presseau | Will lead knowledge translation studies (interview and survey studies). |
| Kednapa Thavorn | Will lead early economic analysis. |
| Robert Holt | Design and synthesis of CD19-CAR constructs and viral vectors, and laboratory-scale validation of CD19-CAR transduced T cells (manufacturing). |
| Harry Atkins | Will advise on clinical protocol and clinical trial design. |
| Raewyn Broady | Will advise on clinical protocol and clinical trial design. |
| Matthew Seftel | Integrated Knowledge User: Physician Partner |
| Patrick Bedford | Integrated Knowledge User: Regulatory Expertise |
| Sasha Van Katwyk | Research Associate (early economic analysis) |
| Emma Grigor | Research Assistant (systematic reviews) |
| Neil Wesch | Research Assistant |
| Casey Lansdell | Laboratory Technician |
| Josh Montroy | Research Associate (systematic reviews and retrospective cohort study) |
| Sarah Asad | Research Coordinator (knowledge translation interview studies) |
| Gisell Castillo | Research Coordinator (knowledge translation interview studies) |
| Madison Foster | Research Assistant (knowledge translation interview studies) |

# GO-CART Reporting Relationships

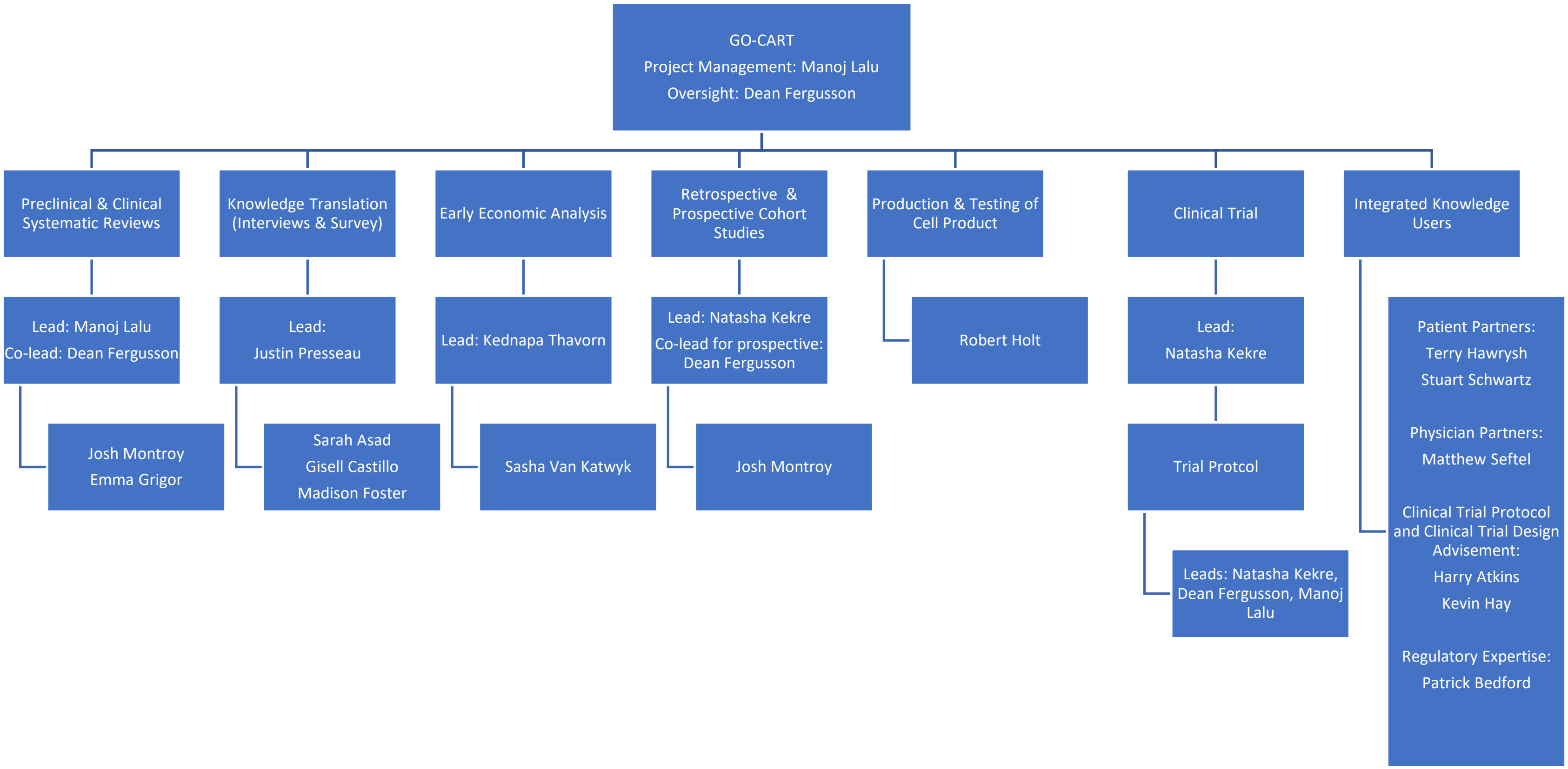

Supplement: Supplementary file 3 — Additional file 3. Terms of Reference. [file 40900_2020_230_MOESM3_ESM.pdf]
